# Equilibrium Propagation for Memristor-Based Recurrent Neural Networks

**DOI:** 10.3389/fnins.2020.00240

**Published:** 2020-03-24

**Authors:** Gianluca Zoppo, Francesco Marrone, Fernando Corinto

**Affiliations:** Department of Electronics, Politecnico di Torino, Turin, Italy

**Keywords:** artificial neural network, biologically plausible learning rule, neuromorphic computing, recurrent neural network, associative memory, memristor

## Abstract

Among the recent innovative technologies, memristor (memory-resistor) has attracted researchers attention as a fundamental computation element. It has been experimentally shown that memristive elements can emulate synaptic dynamics and are even capable of supporting spike timing dependent plasticity (STDP), an important adaptation rule that is gaining particular interest because of its simplicity and biological plausibility. The overall goal of this work is to provide a novel (theoretical) analog computing platform based on memristor devices and recurrent neural networks that exploits the memristor device physics to implement two variations of the backpropagation algorithm: recurrent backpropagation and equilibrium propagation. In the first learning technique, the use of memristor–based synaptic weights permits to propagate the error signals in the network by means of the nonlinear dynamics via an analog side network. This makes the processing non-digital and different from the current procedures. However, the necessity of a side analog network for the propagation of error derivatives makes this technique still highly biologically implausible. In order to solve this limitation, it is therefore proposed an alternative solution to the use of a side network by introducing a learning technique used for energy-based models: equilibrium propagation. Experimental results show that both approaches significantly outperform conventional architectures used for pattern reconstruction. Furthermore, due to the high suitability for VLSI implementation of the equilibrium propagation learning rule, additional results on the classification of the MNIST dataset are here reported.

## Introduction

In the last few decades, the search of innovative computing platforms that could offer new, ultra-low power processing methods and architectures has intensified. Neuromorphic computing approaches aim to go beyond the state-of-the-art in conventional digital processing by exploiting complex dynamics and nonlinear phenomena emerging from the physics of nonvolatile memory devices (e.g., memristors) (Chua, 1971; Strukov et al., 2008). The hallmark of this kind of devices is the peculiar analog signal storing capability that allows them to mimic the behavior of neural synapses. The processing is not only analog and different from current digital processors, but also enhances computing speed and power efficiency for large sets of sensor data. This has been achieved by combining memristor technology with advanced deep learning algorithms used to train neural networks. In supervised learning, one of the most popular method used for training feedforward neural networks is the backpropagation algorithm. Although it is considered a powerful technique, it is computationally expensive and is commonly labeled as biologically implausible. The generalization of this rule to continuous-time recurrent networks was first introduced by Almeida (1987) and Pineda (1988) who independently obtained the same results. Recurrent backpropagation aims to iteratively adjust the weight matrix of the network in order to let the system converge, for fixed input and initial state, to a desired attractor. As for feedforward neural networks, this is achieved by minimizing a particular loss function associated to the system parameters with the difference that the error signal is now backpropagated by introducing an associated differential equation. This allowed to avoid the direct gradient's computations and reduced the large number of required multiplications. However, the necessity of a side network for the propagation of error derivatives makes this technique still highly different from emulating the brain complex computation. This hypothesis is further supported by the fact that there is no known mechanism that could explain how an error message is propagated backwards through the same pathway of the incoming signal. Recently, Scellier and Bengio (2017) proposed an alternative solution to the use of a side network by introducing Equilibrium Propagation, a learning technique used for energy-based models. The advantage of this approach is indeed the requirement of just one kind of neural computation for the training phase of the network. Firstly, inputs are clamped and the network relaxes to a fixed point which corresponds to a local minimum of the energy function. Secondly, after introducing a small external error signal, the network relaxes to a new but close-by fixed point which now corresponds to a rather lower cost value. Even though the two methods seem quite different, it is easy to observe that both share the same goal, finding low-energy configurations that have low cost values. The aim of this work is to propose a novel (theoretical) analog computing platform based on memristor devices and recurrent neural networks that exploits the memristor device physics to implement two variations of the backpropagation algorithm. In the first section, it is provided a brief introduction on memristors and their peculiar properties useful for the physical implementation. In the second section, a general introduction on biological algorithms is presented with particular attention on recurrent backpropagation and equilibrium propagation. In the last section, the two techniques are compared with the existing algorithms used in pattern reconstruction providing results of their compelling efficiency. Lastly, it is shown the application of a memristor-based recurrent neural network trained with equilibrium propagation used for the classification of a small subset of the MNIST dataset. The choice of using only this learning rule was mainly dictated by the fact that using a side network, as in the recurrent backpropagation approach, would at least double the required IC area.

## Memristor–Based Recurrent Neural Network

Massive progress has already been made with neuromorphic systems based on traditional analog and digital integrated circuits. Among all the recent alternatives which aim to emulate neurobiological components and functions, memristive devices have drawn particular attention (Jo et al., 2010). Memristors, often termed as Resistive Switching devices, are single-port electrical dynamical systems whose conduction properties depend on the history of applied input at the port (Chua and Sung Mo, 1976). The typical memristor physical implementation consists of two metal electrodes sandwiching a switching material. An intuitive connection links these two electrodes to the corresponding role of axons and dendrites and the switching layer to the variable interconnection weight of synapses. The crossbar architecture is probably the most commonly used computing structure exploiting the memristive behavior for mapping neural networks in hardware. Its basic working principle is the application of Kirchhoff's Current Law to compute the input to the i-*th* neuron as the algebraic sum of the weighted inputs $I_{i}=\sum_{j}G_{ij}v_{j}$. Here, *I*_*i*_ is the *i*-th input current, *G*_*ij*_ is the connecting memductance between the *i*-th and *j*-th neurons and *v*_*j*_ is the output voltage generated by the *j*-th neuron. This produces the vector-matrix multiplication *in situ* by a single read operation which eliminates the need for constant bidirectional data transfer from the memory to the computing unit (Sun et al., 2019).

The most peculiar characteristic of this kind of devices is the synaptic plasticity effect which is also observed in biological neural systems. Since conductances can be tuned by controlling the coordinated activity of pre- and post-synaptic neurons, memristor-based neural networks can consequently emulate neurobiological phenomena while mimicking the underlying learning process. From neurological studies, it turned out that the neural coding is highly dynamic, therefore recurrent neural networks seem well suited to model similar behavior and have been used to investigate the mechanisms adopted by neurons populations in solving various complex tasks.

For this reason, consider a recurrent neural network and let each synaptic weight be described by a generic memristor (see also Corinto et al., 2015; Leon, 2015) that satisfies the following equations:

(1) $$\begin{array}{l} \{\begin{array}{l} i=G(x)v\\ \frac{dx}{dt}=f(x,v) \end{array} \end{array}$$

where *i* is the current, *v* is the voltage, *G*(·) is the memductance and **x** is the internal state vector. Let the memristor–based synaptic weight be *G*(**x**) = *w*, with the purpose of giving a formal description of the network's learning process, the state vector can be defined by the two following dynamics:

(2) $$\{\begin{array}{l} i=wv\\ \frac{dw}{dt}=f_{1}(w,v,y)\\ \frac{dy}{dt}=f_{2}(w,v,y) \end{array}\;\{\begin{array}{l} i=wv\\ \frac{dw}{dt}=g(w,v) \end{array}$$

In the next sections, the derivation of the previously mentioned variants of the backpropagation algorithm for recurrent neural network is given in order to clarify the use of the different choice of the state vector **x**. Further work is still needed to find physical devices that approximate the proposed memristive synapse dynamics in (2). Many models of memristor devices (e.g., Phase Change Memory, Resistive NonVolatile Memory, etc.) have been presented during the last decade but unfortunately the existing mathematical representations are not suitable for this kind of investigation. The current approach available in literature is to embed memristor device in suitable synaptic circuit so that the dynamics of internal (state) variables can be controlled by appropriate pulses. Thus, the learning rules can be implemented by a series of discrete programming pulses that perform the weights update according to the learning rules defined by the recurrent backpropagation and the equilibrium propagation algorithms. This can be obtained by means of amplitude/duration modulation of a voltage (or current) pulse applied on a physical device via the 1T–1R (one transistor–one memristor) architecture (see Liu et al., 2015; Merced-Grafals et al., 2016). An alternative approach is based on the use of emulator of generic memristors (Ascoli et al., 2016; Assaf et al., 2019) such that the dynamics in (2) can be obtained. Although the physical realization of memristor synapses is a challenging problem, its investigation is out of the scope of the present work that aims to show how memristor–based recurrent neural networks with memristor synapses support equilibrium propagation algorithms. A further study will be devoted to tackle the implementation of proposed memristor synapses.

## Biologically-Plausible Learning Algorithms

The rules that govern the learning process in the brain are poorly understood. Despite the great success of deep learning in a wide variety of complex tasks (LeCun et al., 2015), learning rules in the brain are most likely local and strictly feedforward. Theoretical analysis of biological neural networks showed indeed that connections between neurons are mostly strengthened depending on the coordinated activity of pre-synaptic and post-synaptic cells rather than computations of all downstream neurons (Hebb, 1949; Gerstner et al., 2014). Therefore nowadays, there is an increasing interest in machine learning and computational neuroscience in the study of neuron-like architecture with local learning rules that aim to approximate the surprising efficiency of the backpropagation training process. Many bio-plausible approaches include feedback allignment (Lillicrap et al., 2016), target propagation algorithms (Lee et al., 2015), membrane potential based backpropagation algorithm (Lee et al., 2016), equilibrium propagation (Scellier and Bengio, 2017), etc. See for example Whittington and Bogacz (2019) for an extensive review. Since neurological research suggests that the neural representation is highly dynamic, models based on recurrent neural networks seem well suited to capture similar behavior and therefore have been used to investigate the mechanisms by which neural populations solve various computational problems. In order to take advantage of the intrinsic nonlinear dynamics of the system, two learning techniques for continuous time recurrent neural networks were mainly considered: recurrent backpropagation and equilibrium propagation. Even though the latter shows a more suitable affinity for VLSI implementations (Scellier and Bengio, 2017), the former represents the first attempt in approaching energy-based models from a supervised point of view and therefore is worth being mentioned and compared. In the next subsections, it is provided a brief introduction to the construction and derivation of both algorithms.

### Recurrent Backpropagation

Consider a Recurrent Neural Network (RNN) whose state vector **v** evolves according to:

(3) $$\begin{array}{l} \frac{dv_{i}}{dt}=-v_{i}+g_{i}\left(\overset{N}{\underset{j=1}{\sum }}w_{ij}v_{j}+I_{i}\right),\;i=1,\ldots ,N \end{array}$$

where *N* is the number of neurons of the network and *I*_*i*_ is an external input to the *i*-th neuron. There is no restriction on the choice of the activation function *g*_*i*_ as long as it is monotone and differentiable (Pineda, 1988). In the most general case, neurons can be considered either as input, output or hidden units depending on the application. The goal of the algorithm is to adjust the weights *w*_*ij*_ so that, for a given initial condition ${\text{v}}^{0}=\text{v}\left(t_{0}\right)$ and a given vector of input **I**, the RNN (3) converges to a desired fixed point ${\text{v}}^{\infty }=\text{v}\left(t_{\infty }\right)$. This is obtained by minimizing a loss function *E* which measures the euclidean distance between the desired fixed point and the actual fixed point:

(4) $$\begin{array}{l} E=\frac{1}{2}\overset{N}{\underset{i=1}{\sum }}J_{i}^{2}=\frac{1}{2}\overset{N}{\underset{i=1}{\sum }}{\left(T_{i}-v_{i}^{\infty }\right)}^{2} \end{array}$$

where *T*_*i*_ is the *i*-th desired output state component and *J*_*i*_ is the *i*-th component of the difference between the current fixed point $v_{i}^{\infty }$ and the target point *T*_*i*_. Observe that *E* depends on the weight matrix **W** through the fixed point **v**^∞^(**W**, **I**). Therefore, one way to drive the system to converge to a desired attractor is to let it evolve in the weight parameter space along trajectories which have opposite direction of the gradient of *E*:

(5) $$\begin{array}{l} \frac{dw_{ij}}{dt}=-\eta \frac{\partial E}{\partial w_{ij}}=\eta \overset{N}{\underset{k=1}{\sum }}J_{k}\frac{\partial v_{k}^{\infty }}{\partial w_{ij}},\;\eta >0 \end{array}$$

where η is the learning rate. The derivative of $v_{k}^{\infty }$ with respect to *w*_*ij*_ is derived by observing that the fixed points of (3) must satisfy the nonlinear equation:

(6) $$\begin{array}{l} v_{k}^{\infty }=g_{k}\left(\overset{N}{\underset{s=1}{\sum }}w_{ks}v_{s}^{\infty }+I_{k}\right). \end{array}$$

Differentiating (6) with respect to *w*_*ij*_ one obtains (for more details see the Appendix):

(7) $$\begin{array}{l} \frac{\partial v_{k}^{\infty }}{\partial w_{ij}}={\left(\delta_{ki}-g_{k}^{\prime }\left({Î}_{k}^{\infty }\right)w_{ki}\right)}^{-1}g_{i}^{\prime }\left({Î}_{i}^{\infty }\right)v_{j}^{\infty } \end{array}$$

where δ_*ki*_ is the kronecker delta. Unfortunately, (7) requires the computation of a reciprocal for computing the weights' update and therefore Pineda (1988) bypassed this problem by considering

(8) $$\begin{array}{l} y_{i}=g_{i}^{\prime }\left({Î}_{i}^{\infty }\right)\overset{N}{\underset{k=1}{\sum }}J_{k}{\left(\delta_{ki}-g_{k}^{\prime }\left({Î}_{k}^{\infty }\right)w_{ki}\right)}^{-1} \end{array}$$

which can be seen as the steady state of the following side network:

(9) $$\begin{array}{l} \frac{dy_{k}}{dt}=-y_{k}+g_{k}^{\prime }\left({Î}_{k}^{\infty }\right)\left(\overset{N}{\underset{i=1}{\sum }}w_{ik}y_{i}+J_{k}\right). \end{array}$$

In conclusion, the weights' update rule is defined by:

(10) $$\begin{array}{l} \frac{dw_{ij}}{dt}=\eta y_{i}^{\infty }v_{j}^{\infty } \end{array}$$

which is therefore dependent on the corresponding fixed points of the dynamical systems (3) and (9). Here is the summary of the whole learning process:

1. Firstly, (3) evolves starting from a random initial condition and converges to the corresponding fixed point **v**^∞^;
2. Secondly, (9) evolves starting again from a random initial condition and converges to the corresponding fixed point **y**^∞^;
3. Lastly, the weights of the matrix **W** are updated according to
  (11) $$\begin{array}{l} \Delta w_{ij}=\eta y_{i}^{\infty }v_{j}^{\infty },\;\eta >0. \end{array}$$

### Equilibrium Propagation

Consider now the following energy function *E*:

(12) $$\begin{array}{l} E\left(\text{v}\right)=\overset{N}{\underset{i=1}{\sum }}\frac{v_{i}^{2}}{2}-\frac{1}{2}\overset{N}{\underset{i,j=1}{\sum }}w_{ij}g_{i}\left(v_{i}\right)g_{j}\left(v_{j}\right)-\overset{N}{\underset{i=1}{\sum }}I_{i}g_{i}\left(u_{i}\right) \end{array}$$

where *N* is the number of neurons of the network and *I*_*i*_ is an external input to the *i*-th neuron. Again, there is no restriction on the choice of the activation functions *g*_*i*_(·)∀*i* = 1, …, *N* as long as they are differentiable and monotone. Assume that the time evolution of the state variable **v** is governed by the gradient dynamics:

(13) $$\begin{array}{l} \frac{dv_{i}}{dt}=-\frac{\partial E}{\partial v_{i}}=-v_{i}+g_{i}^{\prime }\left(v_{i}\right)\left(\overset{N}{\underset{j=1}{\sum }}w_{ij}g_{i}\left(v_{j}\right)+I_{i}\right),\;i=1,\ldots ,N \end{array}$$

Observe that, the network is recurrently connected with symmetric connections (i.e., *w*_*ij*_ = *w*_*ji*_). Typically in the supervised learning framework, the output units aim to recreate their targets **T**. The deviation of the fixed points **v**^∞^, output values of the network, from the targets **T** is measured by the quadratic loss function:

(14) $$\begin{array}{l} C=\frac{1}{2}\overset{N}{\underset{i=1}{\sum }}{\left(T_{i}-v_{i}\right)}^{2} \end{array}$$

Observe that this function is defined for any state of **v**. The central idea of Equilibrium Propagation is to introduce the augmented energy function:

(15) $$\begin{array}{l} F\left(\text{v},\text{W},\text{T}\right)=E\left(\text{v},\text{W}\right)+\beta C\left(\text{v},\text{W},\text{T}\right)\\ \;\text{v},\text{T}\in {\mathbb{R}}^{N},\text{W}\in {\mathbb{R}}^{N\times N},\beta \geq 0 \end{array}$$

and replace the free dynamics with the augmented dynamics:

(16) $$\begin{array}{l} \frac{dv_{i}}{dt}=-\frac{\partial F}{\partial v_{i}} \end{array}$$

Here, the second term $-\beta \frac{\partial C}{\partial v_{i}}$ gradually pushes **v** toward configurations that have lower cost values. This is done, as in the previous model, by simply adjusting **W** so as to minimize the cost value of the fixed point. Now, in order to derive the corresponding learning rule, let us introduce the following objective function

(17) $$\begin{array}{l} J\left(\text{W}\right)=C({\text{v}}^{\infty },\text{W},\text{T})\;\text{v},\text{T}\in {\mathbb{R}}^{N},\text{W}\in {\mathbb{R}}^{N\times N} \end{array}$$

Observe that *J*(**W**) is the cost at the fixed point. The equilibrium propagation algorithm estimates the gradient $\frac{\partial J}{\partial \text{W}}$ based on measures at the fixed points of the free and the augmented dynamics that we will set as **v**^∞^ and ${\text{v}}_{\beta }^{\infty }$. Scellier and Bengio (2017), indeed, proved the following statement:

(18) $$\begin{array}{l} \frac{\partial J}{\partial \text{W}}=\underset{\beta \to 0}{\lim}\frac{\frac{\partial F}{\partial \text{W}}\left({\text{v}}_{\beta }^{\infty }\right)-\frac{\partial F}{\partial \text{W}}\left({\text{v}}^{\infty }\right)}{\beta } \end{array}$$

offering an alternative way to estimate the gradient of the objective function. Therefore, the network follows the following dynamics for the training phase:

1. Firstly, **T** is clamped and the network follows the free dynamics (13) relaxing to the free fixed point **v**^∞^ where $\frac{\partial F}{\partial \text{W}}\left({\text{v}}^{\infty }\right)$ is measured (free phase);
2. Secondly, the influence parameter is introduced and the network relaxes to a new but nearby fixed point ${\text{v}}_{\beta }^{\infty }$ where $\frac{\partial F}{\partial \text{W}}\left({\text{v}}_{\beta }^{\infty }\right)$ is measured (weakly clamped phase).
3. Lastly, the weights of the matrix **W** are changed according to (18) and updated as follows:
  (19) $$\begin{array}{l} \Delta w_{ij}\propto +\;\eta \left[g_{i}\left(v_{i}^{\beta ,\infty }\right)g_{j}\left(v_{j}^{\beta ,\infty }\right)-g_{i}\left(v_{i}^{\infty }\right)g_{j}\left(v_{j}^{\infty }\right)\right],\;\eta >0. \end{array}$$

## Implementation and Experimental Results

In this section, it is first provided an experimental evidence of the two models' efficiency in a pattern reconstruction task. Afterwards, an example on the classification of a subset of MNIST dataset is here reported using equilibrium propagation as learning rule.

### Pattern Reconstruction

**Figure 1:**
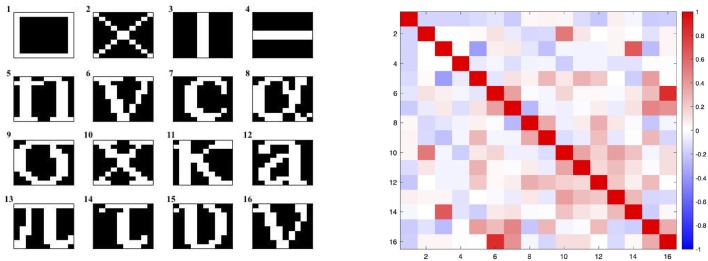
In the left panel, the dataset of all the 16 patterns to be learnt. In the right panel, a graphical representation of the corresponding patterns' correlation matrix computed with the Pearson correlation coefficient.

In this section, a comparison between the two aforementioned training algorithms for pattern's reconstruction task is presented. For this kind of application, input units are chosen to be simultaneously output units and no hidden units are considered. Moreover, due to the construction of the gradient dynamics (13), symmetric weights were chosen for both methods. This condition also guarantees the convergence of the model (3). During the training phase, each image shown in Figure 1 is repeatedly proposed to the network by means of a constant input **I** until it is memorized. In the case of multiple patterns to be learnt, the previous steps are performed for each single image of the dataset for different epochs. Here patterns were shown to the network in the same order for each epoch but this choice was not restrictive since similar performances were obtained even in the case the images were proposed in a random fashion. In order to train the network, the following hyperparameters and initial conditions were set for the training phase:

- Random initialization of the state variable **v**;
- In the recurrent backpropagation case, each single time the first state variable converges, the second variable is reset to **y**(0) = (0.5, …, 0.5)^*T*^ ∈ ℝ^*N*^;
- The matrix **W** is symmetric and initialized with uniform random values between [−0.1; 0.1];
- The activation functions *g*_*i*_ ∀*i* = 1, …, *N* are hyperbolic tangent functions;
- The learning parameter η = 0.01;
- The number of epoch is 300.
- Time spans for the simulation of the dynamics systems are chosen in order to guarantee the convergence of the state variables.

**Figure 2:**
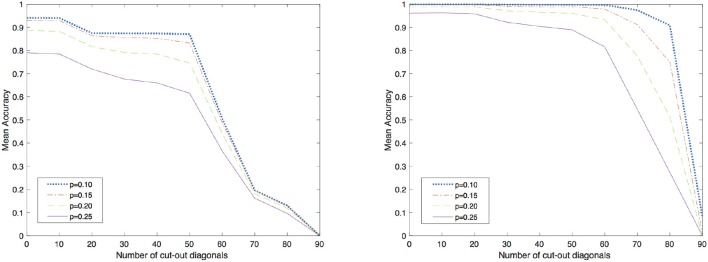
Accuracy for different radius of connectivity. On the left, the results obtained by using Recurrent Backpropagation and on the right, the results obtained by using Equilibrium Propagation. *p* is the probability of flipping each pixel of the image from white to black and viceversa.

**Figure 3:**
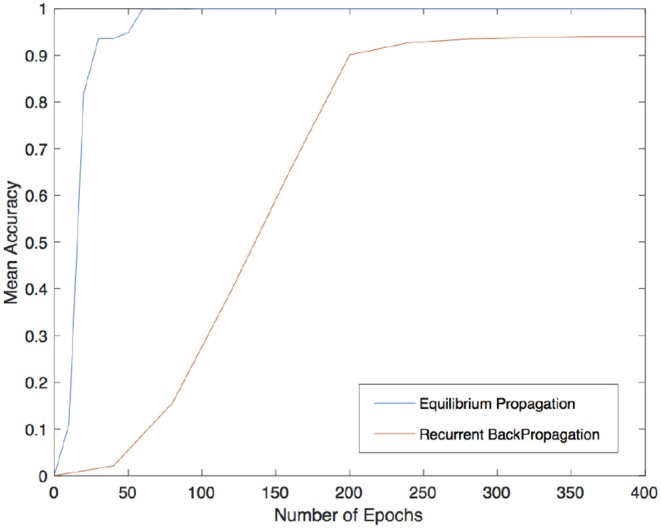
Mean accuracy over 1000 reconstructed patterns for different number of epochs using Equilibrium Propagation (in blue) and Recurrent Backpropagation (in orange).

With the aim of assessing the applicability of this method in VLSI implementation, a short analysis on the importance of local-global connections of the network's neurons was performed. For further details on the relation of the topology and the computational performance of attractor neural networks refer to McGraw and Menzinger (2003), Hasler and Marr (2013) or Stauffer et al. (2003), Tanaka et al. (2019) for additional results on current approaches for enhancing the energy efficiency of hardware-level neural networks by means of sparse and less costly number of connections. Here, for sake of simplicity, a simpler investigation was carried out by increasingly disconnecting global connections arising from a full matrix by simply setting to zero all the elements that were located outside a band about the main diagonal. In order to test the network, corrupted patterns were created by flipping, with probability *p*, each pixel of the image from white to black and viceversa. The cut of *K* outer diagonals from the matrix reduces the number of synapses from *N*^2^ to *N*^2^ − *K*(*K* + 1). In this analysis, a corrupted pattern is recognized as reconstructed if the least square error with respect to the original images is equal to 0. The validation was carried out by testing the recovery capabilities of the network against 5000 corrupted patterns for each class shown in Figure 1. The results obtained by both methods are shown in Figure 2 with different levels of test images' corruption (e.g., *p* = 0.10, *p* = 0.15, *p* = 0.20, and *p* = 0.25). It is easy to see that both methods seem to reach promising and equally meaningful results in the case of fully connected networks. However, Equilibrium Propagation is able to get better results even with a small amount of connections. This fact, together with the absence of a side network really motivates us to investigate this method as a solution worth to be considered for a VLSI implementation. This improvement might be induced by the noisy estimator of the gradient given by (19) that helps the network to efficiently explore the parameter space by avoiding to get stuck in local minima. This might be further seen in Figure 3 where good values of accuracy are already obtained by Equilibrium Propagation in the first 50 epochs whereas Recurrent Backpropagation needs at least 300 epochs. In last analysis, in order to assess the efficiency of the two novel methods, it is additionally performed a comparison with two of the most used learning rules for training networks in associative memory's tasks. It is well known that a standard Hopfield model trained on uncorrelated patterns with the Hebbian rule has an approximate capacity of 0.14*N* (*N* is the number of units in the network) (McEliece et al., 1987). Unfortunately, this capacity decreases significantly if patterns are correlated. To overcome this problem, a novel learning method has been introduced by Storkey (1997). The Storkey learning rule presents indeed a significantly improved performance over the standard Hopfield model, both with correlated and uncorrelated data.

**Figure 4:**
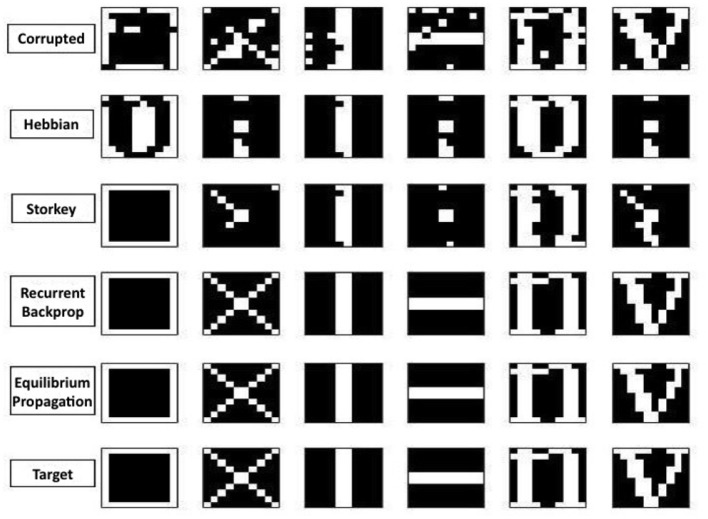
From the top row: six corrupted patterns with probability *p* = 0.1, reconstructed pattern with hebbian rule, Storkey rule, recurrent backpropagation rule, Equilibrium Propagation rule and in the last row the target patterns.

However, as shown in Table 1 and in the examples of Figure 4, the results provide evidence that both recurrent backpropagation and the equilibrium propagation algorithms are perfectly able to reconstruct even in the presence of correlated patterns.

**Table 1:** Accuracy for each single learning rule over 1,000 corrupted images, with probability 0.1, for each of the 16 classes.

	**Hebbian rule**	**Storkey rule**	**Recurrent BackProp rule**	**Equilibrium Propagation rule**
Accuracy	0.1792	0.2663	0.9968	0.9971

### Pattern Classification

As a second experimental result, it is now provided an application of the model introduced by Scellier and Bengio (2017) in a pattern classification task of a subset of the MNIST dataset: 5 classes and 600 patterns for each class. The model used here is still a recurrent neural network with symmetric connections, 1 hidden layer, no skip-layer and no lateral connections. Following Scellier and Bengio (2017), a hard sigmoid was chosen as activation function and the training process was performed by iterating the successive steps:

1. Fix the pattern as a constant input;
2. Run the free phase until convergence of the hidden and the outputs units may be reached and collect $g\left(v_{i}^{\infty }\right)g\left(v_{j}^{\infty }\right)$;
3. Run the weakly clamped phase until convergence and collect $g\left(v_{i}^{\beta ,\infty }\right)g\left(v_{j}^{\beta ,\infty }\right)$;
4. Update the synaptic weights according to (19).

In order to perform the training process, (16) was first discretized into short time lapses of duration ϵ as follows:

(20) $$\begin{array}{l} v_{t+1}=v_{t}-\epsilon \frac{\partial F}{\partial v_{i}} \end{array}$$

**Figure 5:**
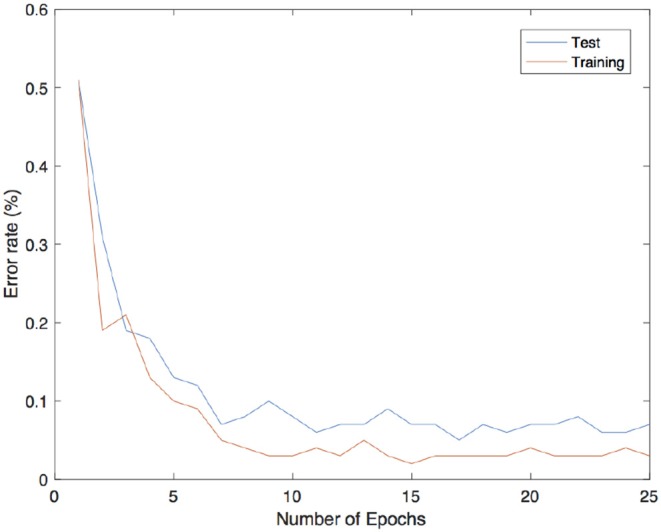
Error rates of the trained neural network over 100 random patterns chosen among the training set (in orange) and 100 patterns from the test set (in blue) using Equilibrium Propagation learning rule.

However, as suggested by Scellier and Bengio (2017), the state variable should be bounded between 0 and 1 and therefore a slightly different update rule was used:

(21) $$\begin{array}{l} v_{t+1}=g\left(v_{t}-\epsilon \frac{\partial F}{\partial v_{i}}\right) \end{array}$$

where *g*(·) is the hard sigmoid function. The predicted value corresponds to the index of the output units which reached the maximum value among all the others. All the hyperparameters chosen were in accordance with the suggestions proposed in Scellier and Bengio (2017): the learning rate ϵ = 0.5 is used for the iterative inference, β = 1 is the value of the clamping factor in the second phase, α_1_ = 0.1, α_2_ = 0.05 are the two different learning rates for updating the parameters in the first and second layer. Observe that the authors were not considering a single learning rate η as in (19). However, instead of choosing a random sign for β for the second phase, the two learning parameters α_1_, α_2_ were decreased by half after each epoch. The results are shown in Figure 5 and are consistent with the findings described in Scellier and Bengio (2017).

## Conclusions

In this paper, the dynamics of memristor–based recurrent neural networks has been analyzed. The network is trained by using two different generalizations of the backpropagation algorithm adapted to the continuous domain and energy-based models. Such *in situ* training learning rules permit to the memristor–based neural network to continuously adapt and adjust the synaptic weights without the direct computation of the loss function's gradient. Although, further work is still necessary to find physical memristor devices/emulators approximating the proposed memristive synapse dynamics, this manuscript provides two learning rules for the weights' update that can be implemented by a series of discrete programming pulses. Simulated results make clear that both methods significantly outperform conventional approach used for pattern reconstruction. In addition, promising results are also obtained by using equilibrium propagation in performing classification tasks.

## Data Availability Statement

Publicly available datasets were analyzed in this study. This data can be found here: http://yann.lecun.com/exdb/mnist/.

## Author Contributions

All authors listed have made a substantial, direct and intellectual contribution to the work, and approved it for publication.

### Conflict of Interest

The authors declare that the research was conducted in the absence of any commercial or financial relationships that could be construed as a potential conflict of interest.
