# Supplementary material for: Equilibrium Propagation for Memristor-Based Recurrent Neural Networks
Source: Front Neurosci. 2020 Mar 24;14:240. doi: 10.3389/fnins.2020.00240 (PMC7105894; doi:10.3389/fnins.2020.00240)
Supplement: Supplementary file 1 [file Presentation_1.pdf]

Appendix

Consider a Recurrent Neural Network (RNN) whose state vector $\mathbf{v}$ evolves according to:

$$\frac{dv_i}{dt} = -v_i + g_i\left(\sum_{j=1}^{N} w_{ij}v_j + I_i\right), \qquad i = 1, \ldots, N \tag{1}$$

where N is the number of neurons of the network and I_i is an external input to the i-th neuron. There is no restriction on the choice of the activation function g_i as long as it is monotone and differentiable [Pineda(1988)]. In the most general case, it is possible to define three different subsets of the network units:

- the subset I of input units;
- the subset O of output units;
- the subset H of hidden units.

The goal of the algorithm is to adjust the weights w_{ij} so that, for a given initial condition $\mathbf{v}^0 = \mathbf{v}(t_0)$ and a given vector of input $\mathbf{I}$, the RNN (1) converges to a desired fixed point $\mathbf{v}^\infty = \mathbf{v}(t_\infty)$. This is obtained by minimizing a loss function E which measures the euclidean distance between the desired fixed point and the actual fixed point:

$$E = \frac{1}{2}\sum_{i=1}^{N} J_i^2 = \frac{1}{2}\sum_{i=1}^{N}(T_i - v_i^\infty)^2 \tag{2}$$

where T_i is the i-th desired output state component and J_i is the i-th component of the difference between the current fixed point v_i^∞ and the target point T_i. Observe that E depends on the weight matrix $\mathbf{W}$ through the fixed point $\mathbf{v}^\infty(\mathbf{W}, \mathbf{I})$. Therefore, one way to drive the system to converge to a desired attractor is to let it evolve in the weight parameter space along trajectories which have opposite direction of the gradient of E:

$$\frac{dw_{ij}}{dt} = -\eta\frac{\partial E}{\partial w_{ij}}, \qquad \eta > 0 \tag{3}$$

where η is the learning rate and must be small enough so that the state variable $\mathbf{v}$ can always be considered to be at steady state [Pineda(1988)]. Computing now the derivatives in (3), one obtains

$$\frac{dw_{ij}}{dt} = -\eta\frac{\partial}{\partial w_{ij}}\left(\frac{1}{2}\sum_{k=1}^{N} J_k^2\right) = -\eta\sum_{k=1}^{N} J_k\frac{\partial J_k}{\partial w_{ij}} = \; = \eta\sum_{k=1}^{N} J_k\frac{\partial v_k^\infty}{\partial w_{ij}} \tag{4}$$

and the derivative of v_k^∞ with respect to w_{ij} is derived by observing that the fixed points of (1) must satisfy the nonlinear equation:

$$v_k^\infty = g_k\left(\sum_{s=1}^{N} w_{ks}v_s^\infty + I_k\right). \tag{5}$$

Differentiating (5) with respect to w_{ij} one obtains:

$$\frac{\partial v_k^\infty}{\partial w_{ij}} = g_k'(\hat{I}_k^\infty)\left[\sum_{s=1}^{N}\frac{\partial w_{ks}}{\partial w_{ij}}v_s^\infty + \sum_{s=1}^{N} w_{ks}\frac{\partial v_s^\infty}{\partial w_{ij}}\right] \tag{6}$$

where $\hat{I}_k^\infty = \left(\sum_{s=1}^N w_{ks} v_s^\infty + I_k\right)$.
Solving (6) in terms of $\frac{\partial v_k^\infty}{\partial w_{ij}}$ and defining $L_{ks} = \delta_{ks} - g_k'(\hat{I}_k^\infty) w_{ks}$ where δ_{ks} is the kronecker delta, it follows that:

$$\begin{cases} \sum_{s=1}^N L_{is} \frac{\partial v_s^\infty}{\partial w_{ij}} = g_i'(\hat{I}_i^\infty) v_j^\infty & k = i \\ \sum_{s=1}^N L_{ks} \frac{\partial v_s^\infty}{\partial w_{ij}} = 0 & k \neq i \end{cases} \tag{7}$$

and therefore for the generic k-th component

$$\frac{\partial v_k^\infty}{\partial w_{ij}} = L_{ki}^{-1} g_i'(\hat{I}_i^\infty) v_j^\infty \tag{8}$$

In conclusion, (3) simply becomes:

$$\frac{dw_{ij}}{dt} = \eta \left[g_i'(\hat{I}_i^\infty) \sum_{k=1}^N J_k L_{ki}^{-1} \right] v_j^\infty . \tag{9}$$

Unfortunately, (9) requires the reciprocal of L_{ki} for computing the weights' update but, considering

$$y_i = g_i'(\hat{I}_i^\infty) \sum_{k=1}^N J_k L_{ki}^{-1} \tag{10}$$

one can avoid this process by introducing an associated dynamical system. Indeed, assuming that $g_i'(\hat{I}_i^\infty) \neq 0 \; \forall \hat{I}_i^\infty \in \mathbb{R}$ and observing that $L_{ki} = L_{ik}$ for construction, (10) is equivalent to:

$$\sum_{k=1}^N L_{ik}^{-1} J_k = \frac{y_i}{g_i'(\hat{I}_i^\infty)} . \tag{11}$$

Solving (11) in terms of J_k one obtains:

$$J_k = \sum_{i=1}^N L_{ik} \frac{y_i}{g_i'(\hat{I}_i^\infty)} . \tag{12}$$

Substituting now the explicit form for L_{ik}, (12) becomes:

$$0 = -y_k + g_k'(\hat{I}_k^\infty) \left(\sum_{i=1}^N w_{ik} y_i + J_k \right) \tag{13}$$

which can be seen as the steady state of the following side network:

$$\frac{dy_k}{dt} = -y_k + g_k'(\hat{I}_k^\infty) \left(\sum_{i=1}^N w_{ik} y_i + J_k \right) . \tag{14}$$

Therefore, the system of differential equations is completely defined by:

$$\frac{dv_i}{dt} = -v_i + g_i \left(\sum_{j=1}^N w_{ij} v_j + I_i \right) \tag{15}$$

$$\frac{dy_k}{dt} = -y_k + g_k'(\hat{I}_k^\infty) \left(\sum_{i=1}^N w_{ik} y_i + J_k \right) \tag{16}$$

$$\frac{dw_{ij}}{dt} = \eta y_i^\infty v_j^\infty \tag{17}$$

Observe that, the weights' update is dependent on the corresponding fixed points of the first two equations.

References

[Pineda(1988)] Pineda, F. J. (1988). Generalization of back propagation to recurrent and higher order neural networks. In *Neural information processing systems*. 602–611
